# Editorial: Is aberrant genome organization a cause or consequence of specific diseases?

**DOI:** 10.3389/fcell.2023.1271790

**Published:** 2023-08-29

**Authors:** Eric C. Schirmer, Joanna M. Bridger

**Affiliations:** ^1^ Institute of Cell Biology, University of Edinburgh, Edinburgh, United Kingdom; ^2^ Centre of Genome Engineering and Maintenance, Biosciences, Department of Life Sciences, College of Health, Medicine and Life Sciences, Brunel University London, Uxbridge, United Kingdom

**Keywords:** genome organization, ageing, lamina associated domains, cancer, laminopathies, epigenetics, DNA repair, lamin A

The past four decades have seen technological advances in the field of genome behaviour that have enabled a pretty clear overview of genome behaviour in healthy cells and how it changes upon external stimuli and during differentiation/senescence. However, ongoing discussions abound regarding mechanisms and functional consequences of gene/chromatin positioning, folding, and dynamics, and how its disruption links with disease. Furthermore, we remain far from completely understanding the involvement of the various nuclear structures, and how even some of these structures are built, let alone their functional, spatial, and temporal association with the genome. There is, however, plenty of evidence and consensus in the field that the nuclear envelope is an immensely important structure with respect to anchoring the genome and the regulation of genome function, not only directly at the nuclear edge but throughout the nucleoplasm. Furthermore, epigenetic control of the genome is partly controlled by nuclear envelope proteins. It is very obvious that genome organization is altered in a wide range of diseases from cancer to tissue degenerative diseases including neural, muscular, skin, metabolic, bone, and fertility, as well as diseases associated with premature ageing. As it has been suggested that most remaining disease alleles to be identified will lie outside of coding regions, to map all genome changes and their impact on gene expression in all diseases will be a major undertaking; therefore, it makes sense to first reflect on the problem to identify underlying common mechanisms for genome organization alterations and establish a defined set of criteria and quality assurances to adhere to, that could also lead to biomarker discovery. This led us to ask the question for this Research Topic. *Is aberrant genome organization a cause or consequence of specific diseases?*

The response yielded the publication of a range of excellent research articles and thoughtful, impactful reviews on epigenetic control and remodelling, the importance of the nuclear envelope and its role in genome organization, and roles of chromatin remodellers for gene regulation; all highlighting best practices needed in the field. Studying genome organization in diseased cells and tissues is not straightforward, as most data are simply correlative, and proper testing requires complex genome-engineered controls.

Some articles were focused more on chromatin remodelling and epigenetics, such as the identification of several new MeCP2 post-translational modifications specifically in the brain that alter its binding kinetics and targeting, which could explain the effects of some Rett syndrome mutations. Here, Schmidt et al. found that R106 that is mutated in Rett syndrome normally becomes di-methylated in the brain and this increases its DNA-binding affinity. A role for the nuclear envelope in chromatin remodelling and epigenetics was made very clear by Marano and Holaska, who demonstrated that emerin at the nuclear envelope interacts with the histone modifiers HDAC3, EZH2, and G9a and altered HDAC3 binding in emerin mutants, resulting in more repressed chromatin at the edge of the nucleus affecting the cell cycle and myogenic differentiation. A comprehensive review on chromatin structural changes in ageing details how ageing genomes suffer histone loss, instability, altered epigenetics, and compositional differences in senescence-associated heterochromatin foci. Sikder et al. also discuss how these alterations to the epigenome and spatial organization as cells age are inherently coupled with cancer progression and differ across the evolutionary landscape.

Evidence for a more direct role of the nuclear envelope in cancer progression was collated by Balaji et al. that involved both nuclear pore complex functions and nuclear membrane structural functions related to 3D-spatial genome organization, oncohistones, and nuclear envelope functions in senescence. Other articles also focused on the nuclear envelope regulation of genome organization. Kervella et al. reviewed how mutations in lamin A causing cardiomyopathy lead to reorganization of nuclear envelope-genome tethers called lamina-associated-domains (LADs) that in turn alter gene expression specifically in cardiomyocytes. They compared data from four different methods to measure genome organization and emphasized the need to apply genome editing tools in patient cells to clarify how genome mis-organization causes disease. Madsen-Østerbye et al. argue the benefits of using combinatorial approaches for 3D-computer modelling of chromosomes and their interactions with the nuclear envelope at LADs to distinguish the most functionally relevant interactions. Importantly, the authors highlight to the field the heterogeneity of these interactions in various cell types, differentiation, senescence, and disease situations where they observed fascinating new patterns.

Finally, other important genome functions linked to the nuclear envelope were considered in two last research articles. Capanni et al. used proximity ligation assays to show that changing pre-lamin A levels are temporally linked to the regulation of early stress responses and DNA repair by 53BP1 that gets recruited to lamin A/C. Todorow et al. focused on a disorder previously not linked to the nuclear envelope, Myotonic dystrophy (DM1). Since DM1 is associated with massive alternative splicing, the authors questioned whether nuclear envelope proteins linked to other muscle disorders are mis-spliced in DM1, finding that structural proteins such as SYNE 1 and 3, SUN1 and 2, and Samp1 were misspliced as well as nuclear membrane proteins involved in musclespecific 3D-genome organization.

Overall, this Research Topic has highlighted the importance of using combinatorial approaches together with some best practices such as genome engineering of patient cells needed to address this important question. It has also shown that there remain previously un-investigated functions that could explain the effects of altered genome organization on specific diseases. Our core Research Topic question of whether aberrant genome organization is a cause or consequence of disease is still unanswered; however, the data argue that where a pathology-causing effect of genome mis-organization has not yet been found, it likely exists and will require scientific creativity like that demonstrated in these Research Topic articles, together with best practices enumerated here to find it.

